# Microparticles from Kidney-Derived Mesenchymal Stem Cells Act as Carriers of Proangiogenic Signals and Contribute to Recovery from Acute Kidney Injury

**DOI:** 10.1371/journal.pone.0087853

**Published:** 2014-02-04

**Authors:** Hoon Young Choi, Sung Jin Moon, Brian B. Ratliff, Sun Hee Ahn, Ara Jung, Mirae Lee, Seol Lee, Beom Jin Lim, Beom Seok Kim, Matthew D. Plotkin, Sung Kyu Ha, Hyeong Cheon Park

**Affiliations:** 1 Division of Nephrology, Department of Internal Medicine, Gangnam Severance Hospital, Seoul, Korea; 2 Severance Institute for Vascular and Metabolic Research, Yonsei University College of Medicine, Seoul, Korea; 3 Department of Internal Medicine, Kwandong University College of Medicine, Gangneung, Korea; 4 Departments of Medicine, Pharmacology and Physiology, New York Medical College, Valhalla, New York, United States of America; 5 Department of Pathology, Yonsei University College of Medicine, Seoul, Korea; 6 Division of Nephrology, Department of Internal Medicine, Severance Hospital, Yonsei University College of Medicine, Seoul, Korea; University of Sao Paulo Medical School, Brazil

## Abstract

We recently demonstrated the use of *in vitro* expanded kidney-derived mesenchymal stem cells (KMSC) protected peritubular capillary endothelial cells in acute renal ischemia-reperfusion injury. Herein, we isolated and characterized microparticles (MPs) from KMSC. We investigated their *in vitro* biologic effects on human endothelial cells and *in vivo* renoprotective effects in acute ischemia-reperfusion renal injury. MPs were isolated from the supernatants of KMSC cultured in anoxic conditions in serum-deprived media for 24 hours. KMSC-derived MPs demonstrated the presence of several adhesion molecules normally expressed on KMSC membranes, such as CD29, CD44, CD73, α4, 5, and 6 integrins. Quantitative real time PCR confirmed the presence of 3 splicing variants of VEGF-A (120, 164, 188), bFGF and IGF-1 in isolated MPs. MPs labeled with PKH26 red fluorescence dye were incorporated by cultured human umbilical vein endothelial cells (HUVEC) via surface molecules such as CD44, CD29, and α4, 5, and 6 integrins. MP dose dependently improved *in vitro* HUVEC proliferation and promoted endothelial tube formation on growth factor reduced Matrigel. Moreover, apoptosis of human microvascular endothelial cell was inhibited by MPs. Administration of KMSC-derived MPs into mice with acute renal ischemia was followed by selective engraftment in ischemic kidneys and significant improvement in renal function. This was achieved by improving proliferation, of peritubular capillary endothelial cell and amelioration of peritubular microvascular rarefaction. Our results support the hypothesis that KMSC-derived MPs may act as a source of proangiogenic signals and confer renoprotective effects in ischemic kidneys.

## Introduction

Microparticles (MPs) are submicron-size vesicles that carry membrane and cytoplasmic constituents of the cells they originated from. They result from plasma membrane remodeling in response to various stimuli, such as oxidative stress or hypoxia. [1], [2] MPs express specific antigens according to their cellular origin. [1], [3] They also act as vectors of growth factors, such as VEGF and proteases, according to the specific stimuli that induced their production. [3], [4]

Cell therapy using pluripotent MSCs to treat kidney injury has been extensively investigated in the past decade.[5]–[9] Initially, it was believed that stem cells home to injured tissue and exert their beneficial effects by differentiating into targets cells and replacing injured tissue. However, it was subsequently demonstrated that transplanted stem cells do not replace injured resident cells but mitigate injury and hasten repair by a paracrine mechanism. [10] MSCs secreted vasculotrophic growth factors such as VEGF, IGF-1, or hepatocyte growth factor and these factors contributed to renal repair. [11] Intravenous infusions of conditioned medium from cultured MSCs consistently exerted renoprotective effects, mimicking the beneficial effects of MSC administration and further supporting the paracrine mechanism of MSC mediated renoprotection. [12] Recent studies have reported that bone marrow MSC- or endothelial progenitor cell-(EPC) derived MPs exert renoprotective effects comparable to those of their origin cells in various experimental acute and chronic renal injuries by inhibiting apoptosis and stimulating proliferation of resident renal cells. [13]–[15]

MPs derived from human bone marrow MSC were found to mediate horizontal mRNA transfer that stimulated proliferation and inhibited apoptosis of tubular epithelial cells *in vitro*. [14] EPC-derived MPs also protected kidneys from acute ischemia-reperfusion (I/R) injury by delivering miRNA that contributed to reprogramming hypoxic resident tubular epithelial and peritubular capillary endothelial cells to a regenerative program. Moreover, EPC-derived MPs protect against chronic kidney damage progression by inhibiting capillary rarefaction, glomerulosclerosis, and tubulointerstitial fibrosis. [16] They showed that EPC-derived MPs were efficiently internalized in both peritubular endothelial cells (TEnCs) and tubular epithelial cells (TEpCs). Moreover, the *in vitro* internalization of EPC-derived MPs within hypoxic TEnCs and TEpCs was followed by reduced apoptosis and enhanced angiogenesis on Matrigel-coated surfaces. [16]

Our previous study showed that a fibroblast-like cell clone isolated from adult mouse kidney differentiated along multiple mesodermal lineages. The kidney-derived MSC (KMSC) migrated to a peritubular interstitial location and expressed interstitial cell markers after subcapsular injection into unilateral I/R injuries in mice kidneys. [17] When *in vitro* expanded KMSC was injected into the tail vein of ischemic mice, they engrafted the ischemic kidney and promoted tubular regeneration and functional recovery from I/R injury. Compared to controls, KMSC injection increased renal tissue VEGF expression and improved peritubular capillary endothelial cell rarefaction. [6]

The aim of the present study was to investigate the characteristics of KMSC-derived MPs and their *in vitro* effects on human endothelial cell viability, apoptosis, and capillary tube formation. Furthermore, the renoprotective effects of KMSC-derived MPs in ischemic mice were evaluated via *in vivo* preservation of peritubular capillary endothelial cells as well as improvement in renal function.

## Materials and Methods

### Mouse Kidney Mesenchymal Stem Cell Cultures and Microparticles Isolations

In our previous report, we isolated and cloned a fibroblast-like cell line from the kidney of an adult FVB/N Tie-2/GFP mouse. [17] The KSMC was cultured on gelatin-coated dishes in minimum essential medium (MEM) with 10% horse serum (Gem Biotech, Woodland, CA, USA) to 80% confluence, as previously described. [17] To generate MPs, KMSC was cultured in a serum-free alpha-MEM in <1% O_2_ using a controlled atmosphere chamber and O_2_ analyzer (Thermo Scientific, Marietta, OH, USA) or KMSC was cultured in a serum-free alpha-MEM with 200 µM hydrogen peroxide and then placed in hypoxic chamber (<1% O_2)_ for 24 hours. Cell debris was removed by centrifugation at 1000 g for 10 min at room temperature. The cell-free supernatants were centrifuged at 50,000 g (Beckman Coulter Optima L-90K, Beckman Coulter, Inc. Brea, CA, USA) for 2 h at 4°C and washed in phosphate-buffered saline (PBS), followed by a second centrifugation in the same conditions. MPs were identified by fluorescence microscopy or FACS analysis and electron microscopy. MPs from KMSC were labeled with PKH26 dye (Sigma) or cell-tracker (Invitrogen) to trace *in vivo* and *in vitro* experiments. MP pellets were suspended in PBS, and FACS measured the number/µl. Endotoxin contamination of MPs was excluded by Limulus testing.

Total MP RNA was extracted using an ExoMir™ Kit according to the manufacturer’s instructions (Charles River Laboratories, Inc., Wilmington, MA, USA). Briefly, supernatants were obtained from KMSC after removing debris by centrifugation at 2000g for 15 min. Supernatant was passed through MP selective filters. The filters were then flushed with an RNA extraction reagent to lyse the captured MPs and release their contents. To abolish the mRNA-dependent effects, MPs were pre-incubated with 1 U/ml RNase (Ambion Inc., Austin, TX, USA) for 1 h at 37°C, as previously described (RNase-treated MPs). [14], [18] The reaction was stopped by adding 10 U/ml RNase inhibitor (Ambion Inc.). The complete degradation of RNA by RNase treatment was confirmed by analyzing total RNA extracted from MPs pre-incubated with RNase on 1% agarose gel (data not shown).

### FACS Analysis of MP

MPs were examined by flow cytometry analyses (BD Canto II, USA). The instrument was rinsed with particle-free rinse solution for 15 minutes to eliminate the background. Different sized beads (Mega-mix, 0.5, 0.9, 3 µm, Biocytex, Marsille, France) represented size markers, and a log scale for forward scatter and side scatter parameters were used for analysis. FACS analysis was performed as described previously [14], [18], [19] using the following FITC- or PE-conjugated antibodies: CD29, CD44 (BD science), CD73, α4 integrin, α5 integrin, and α6 integrin (Biolegend, San Diego, CA). FITC- or PE- mouse non-immune isotypic IgG were used as a control.

### Incorporation of MPs into HUVEC and Peritubular Endothelial Cells

To evaluate the incorporation of MPs into human umbilical vein endothelial cells (HUVEC), MPs were pre-incubated with PKH 26 dye for 30 min at 37°C. The incorporation was studied by FACS analyses and immunofluorescence microscopy. The role of adhesion molecules expressed by KMSC-derived MP surfaces in target cell incorporation was then investigated. MPs were pre-incubated (15 min at 4°C) with blocking antibodies (1 µg/ml) against the identified adhesion molecules such as anti-α4 integrin, anti-α5 integrin, anti-α6 integrin, anti-CD29, CD44, or trypsin (0.5mM, Invitrogen) before incubation with the cells.

To investigate previously reported MSC derived-MP mediated horizontal mRNA transfer, [14], [18] KMSC was transfected with CMV-GFP using lipofectamine (Invitrogen). MPs were isolated from KMSC 48 hours after transfection. HUVEC was incubated with MPs isolated from KMSC transfected with GFP or without GFP. As a positive control, GFP-DNA was used. To investigate *in vivo* MP mediated horizontal mRNA transfer, mice received an intravenous injection of MPs derived from KMSC transfected with GFP or saline alone into the tail vein after I/R injury. RT-PCR analysis was performed on total RNA from HUVEC and kidney tissues. β-actin mRNA was used as a housekeeping gene.

### *In vitro* Cell Proliferation, Apoptosis, and Angiogenesis Assay

HUVEC was seeded in EBM-2 media (Lonza) deprived of FBS into 96-well plates at 5×10^3^ cells/well. Cell proliferation was determined by EZ-Cytox cell proliferation assay kit (Daeil Lab Service Co, Seoul, Korea) according to the manufacturer’s instructions. Briefly, 20 µl of EZ-Cytox kit reagent was added to each cell cultured well of a 96-well microplate and then incubated at 37°C in a humidified CO_2_ incubator for 3 h. After incubation, optical density (OD) was measured at a wavelength of 450 nm using an absorbance microplate reader (BioTek Instruments, Inc., Winooski, VT, USA). Apoptosis was evaluated using an apoptosis kit (Roche, USA) according to the manufacturer’s instructions. In addition to HUVEC, adult dermis human microvascular vein endothelial cells (HMVEC, Life technologies, CA USA) were seeded in Medium 131 (Gibco) into 96-well plates at 5×10^3^ cells/well for apoptosis assay.

*In vitro* tube formation was studied on HUVEC (2×10^4^ cells/well) seeded on growth factor–reduced Matrigel (BD Science). After cells were attached, KMSC-derived MPs (2.5×10^6^, 5×10^6^, 10×10^6^/well) and RNase-treated MPs were added. The cells were observed under a Nikon-inverted microscope (Kanagawa, Japan) after 6-hour incubation at 37°C. The number of tube formation, which was defined as combination of tube number and number of sprouts, was counted by a blinded observer. [20]

### Quantitative Real Time PCR

Quantitative real-time PCR was performed on the total RNA extracted from KMSC and KMSC-derived MPs prepared by culturing KMSC in a microenvironment mimicking AKI.

First-strand cDNA was produced from total RNA using cDNA Synthesis Kit (SuperScript® III First-Strand Synthesis System, Invitrogen Inc.). Briefly, 1–2 µg RNA, 2 µl RT buffer, 1 µl 10mM dNTP mixture, 1 µl random hexamer, 2 µl 0.1M DTT, 1 µl reverse transcriptase, and nuclease-free water were used for each cDNA synthesis. Twenty microliters of RT-PCR mix containing ten µl of 2X SYBR GREEN PCR Master Mix (Applied Biosystems), 100 nM of each primer, and 0.5 µl of MV cDNA were assembled using a Real Time System (ABI 7900 HT). Negative cDNA controls (no cDNA) were cycled in parallel with each run.

The following PCR primers were used for amplification: VEGF 120 forward 5′-CCACGTCAGAGAGCAACATCAC-3′ and reverse5′- GGCTTGTCACATTTTTCTGGCT-3′, VEGF 164 forward 5′-AGGATGTCCTCACTCGGATG-3′ and reverse 5′-AAAGGACTTCGGCCTCTCTC-3′, VEGF 188 forward 5′- GTTCGAGGAAAGGGAAAGGGT-3′ and reverse 5′-GTCTGCGGATCTTGGACAAAC-3′, IGF1 5′- GCT CTG CTT GCT CAC CTT CAC -3′ and reverse 5′-GAA TGC TGG AGC CAT AGC CT- 3′, and FGF forward 5′-GCG ACC CAC ACG TCA AAC TA-3′ and reverse 5′-TCC CTT GAT AGA CAC AAC TCC TC-3′.

### Mice Model of AKI

The animal study protocol was in accordance with guidelines for laboratory animals and approved by the Institutional Animal Care and Use Committee, Department of Laboratory Animal Medicine, Medical Research Center, Yonsei University College of Medicine (2010-0226-1). Adult (8–12 weeks old) FVB/N mice were purchased from Orientbio (Gyeonggi-Do, Korea) and were kept under temperature-controlled conditions of 12-h light/dark cycle, with water and food ad libitum. Mice were anesthetized with intraperitoneal injection of a combination of zoletil (20 mg/kg) and xylazine (10 mg/kg), and all efforts were made to minimize suffering. Bilateral renal ischemia in male FVB/N mice was performed according to a previously detailed protocol. [6] Mice were anesthetized followed by midline laparotomy and bilateral renal pedicle clamping with microserrefines (Fine Science Tools, Foster City, CA, USA). The abdomen was covered with gauze moistened in saline. Mice were well hydrated with saline at a constant temperature using a heated thermoplate (Jeung Do Bio & Plant Co, Korea) during the ischemia period. The clamps were released and reperfusion was visually confirmed after 30 min of ischemia. After I/R injury, mice received an intravenous injection of MPs from KMSC (2 × 10^7^ per mouse), RNase- treated MP, KMSC (1 × 10^6^ per mouse) in 150 µL saline, or saline alone into the tail vein. For each group, mice were killed at day 3 (n = 5 per group) after I/R injury. Kidney tissues were processed for morphologic studies. Blood samples for measuring serum creatinine were collected before, and at 1 and 3 days after I/R injury with vehicle control, KMSC or MP. Creatinine concentrations were determined using a Fuji Dri-Chem 4000i system (Fujifilm, Japan).

### Morphologic Studies

4 µm-thick paraffin kidney sections were stained with PAS for renal histology. Tubular injury was semiquantitatively scored by a renal pathologist (BJ Lim), who was blinded to study design, by assessing the percentage of tubules in the outer medulla and corticomedullary junction that showed tubular dilatation, atrophy or cast formation or sloughing of tubular epithelial cells, and thickening of tubular basement membrane as follows;; 0, none; 1+, <10%; 2+, 10–25%; 3+, 26–45%; 4+, 46–75%; 5+, >75% of tubules. Twenty fields randomly selected from cortex and corticomedullary junction on each slide section were examined at ×400 magnification, as previously reported. [21], [22] Transmission electron microscopy was also performed on MPs or on cultured KMSC releasing MPs. The KMSC-MPs engrafted in peritubular capillaries were detected by colocalizing green fluorescent CellTracker™-labeled MPs within the red fluorescent CD 31 (Abcam) labeled peritubular capillary endothelial cells by confocal microscope (LSM 510, Carl Zeiss, Jena Germany) using × 40 fluorescence objective lens. Green fluorescent CellTracker™ and red fluorescent CD31 double labeled MPs were counted by examining 20 viewing fields randomly selected from the cortex and corticomedullary junction on each slide at ×400 magnification (the number of MPs/mm^2^). Immunohistochemistry was performed to detect the proliferation of peritubular capillary endothelial cells. Kidney sections were subjected to antigen retrieval, and slides were blocked and labeled with 1∶100 of anti-CD 31(Abcam) and 1∶200 of monoclonal anti-PCNA (Millipore). Immunoperoxidase staining was performed using a 1∶100 dilution of anti-rabbit HRP (Abcam) or anti-mouse IgM (Invitrogen). CD31 and PCNA-positive cells were scored by counting the number of positive nuclei per field in 10 randomly chosen sections of kidney cortex at ×400 magnification. Quantification of peritubular capillary loss was determined by the rarefaction index, as previously described. [6] Briefly, CD31-immunostained sections were examined through a 10×10 grid under a × 40 objective. Each square within the grid that did not contain a CD31-positive capillary was counted. At least twenty fields were examined on a cross-section of each kidney, and a mean score per section was calculated. This scoring system inversely reflects peritubular capillary rarefaction, in which low values represent intact capillaries and higher values indicate loss of capillaries. Terminal deoxynucleotidyl transferase-mediated dUTP nick end-labeling (TUNEL) assays were performed using an In Situ Cell Death Detection Kit (Roche, Indianapolis, IN, USA) to detect cell apoptosis in the kidneys. The positive nuclei in the field were examined for under confocal microscope (LSM 510, Carl Zeiss, Jena Germany) using × 40 fluorescence objective lens and analyzed with LSM5 software. Twenty fields randomly selected from cortex and corticomedullary junction on each slide section were examined.

### Statistical Analysis

Data are expressed as mean ± S.E. Differences between groups were analyzed by Mann-Whitney test or Kruskal–Wallis test using SPSS 20.0 (SPSS, Chicago, IL, USA). A P value less than 0.05 was considered statistically significant.

## Results

### Characterization of KMSC-derived MPs

**Figure 1:**
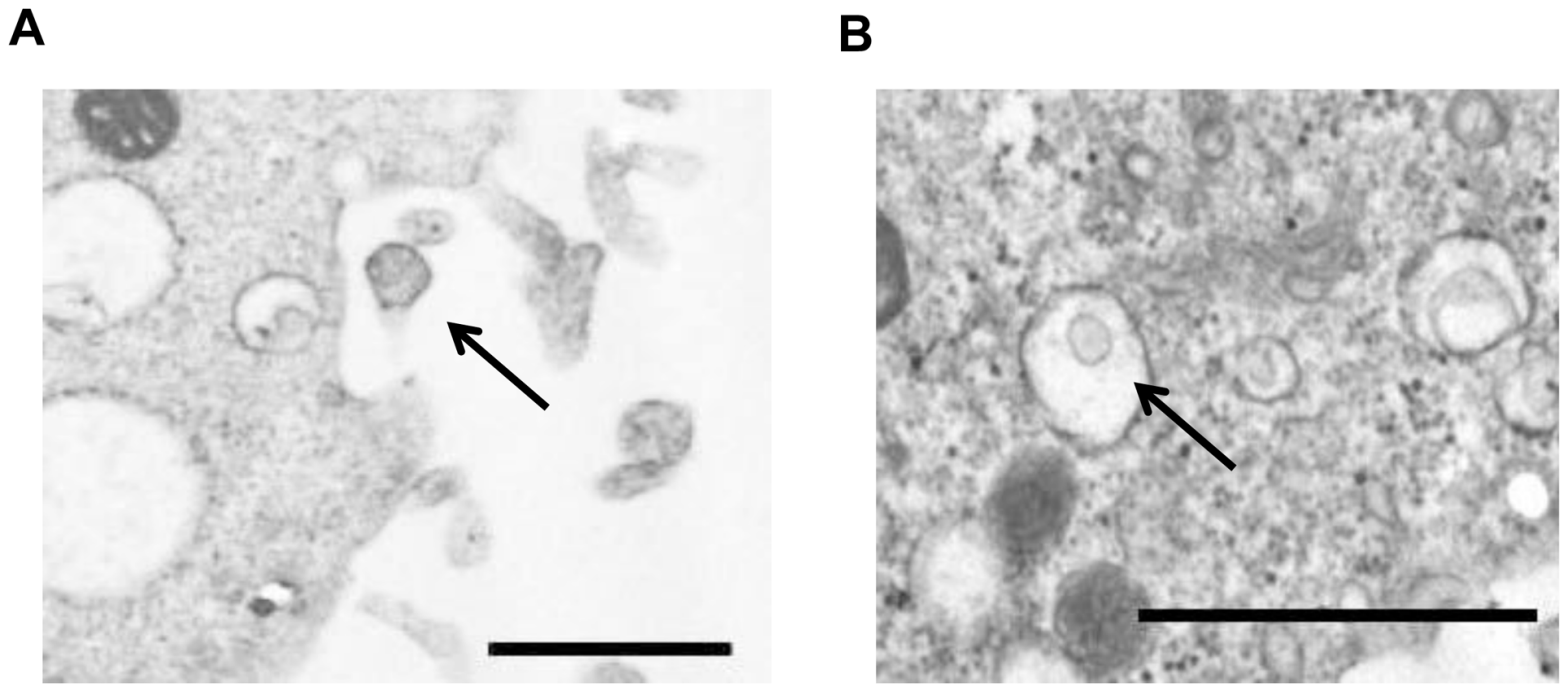
Electron microscopy analyses of KMSC-derived microparticles (MPs). Representative micrographs of transmission electron microscopy obtained upon MP release from the surface of KMSC (A); MPs within larger vesicles in the cell cytoplasm were shown (B). Black bar represents 1000 nm.

**Figure 2:**
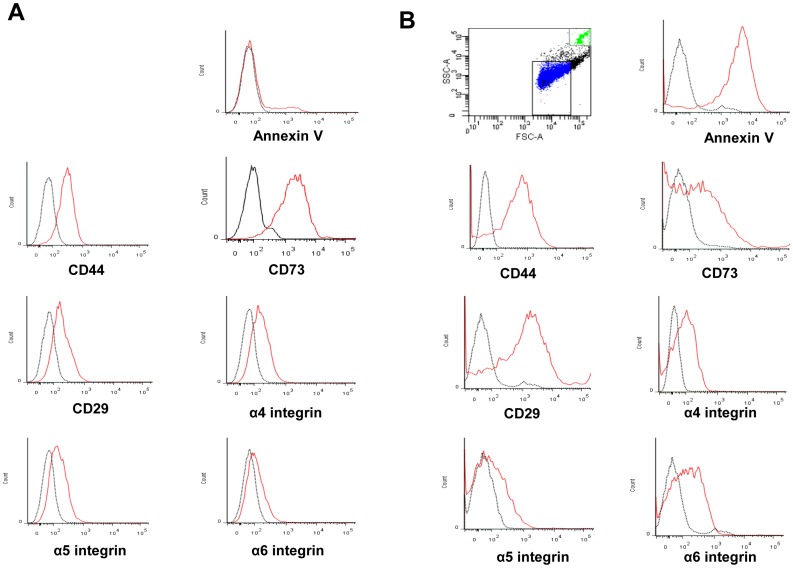
Cytofluorimetric characterization of KMSC (A) and KMSC-derived MPs (B). One µm and 6 µm beads were used as internal size standards. P1 area was compatible with 1 µm. Red lines indicate the expression of annexin V, CD44, CD73, CD29, α4 integrin, α5 integrin, and α6 integrin surface molecules. Black lines indicate isotypic controls.

Transmission electron microscopy demonstrated MPs being released from the cell surface or within larger vesicles in the cytoplasm of KMSC (Figure 1A and B). By flow cytometric analysis, KMSC-derived MPs were mainly detected at a region below the forward scatter signal corresponding to 1 µm beads, which were used as internal size standards (Figure 2B). KMSC-derived MPs positive for Annexin V (86%) and they further expressed CD44, CD73, CD29, α4 integrin, α5 integrin, and α6 integrin, which were also expressed on KMSC plasma membranes (Figure 2B). These findings indicate that MPs express several adhesion molecules of KMSC plasma membranes on their surfaces. The expression of these adhesion molecules was shown to be instrumental in incorporating MPs into target cells.

### Proangiogenic Gene Expression Analysis of KMSC-derived MPs

**Figure 3:**
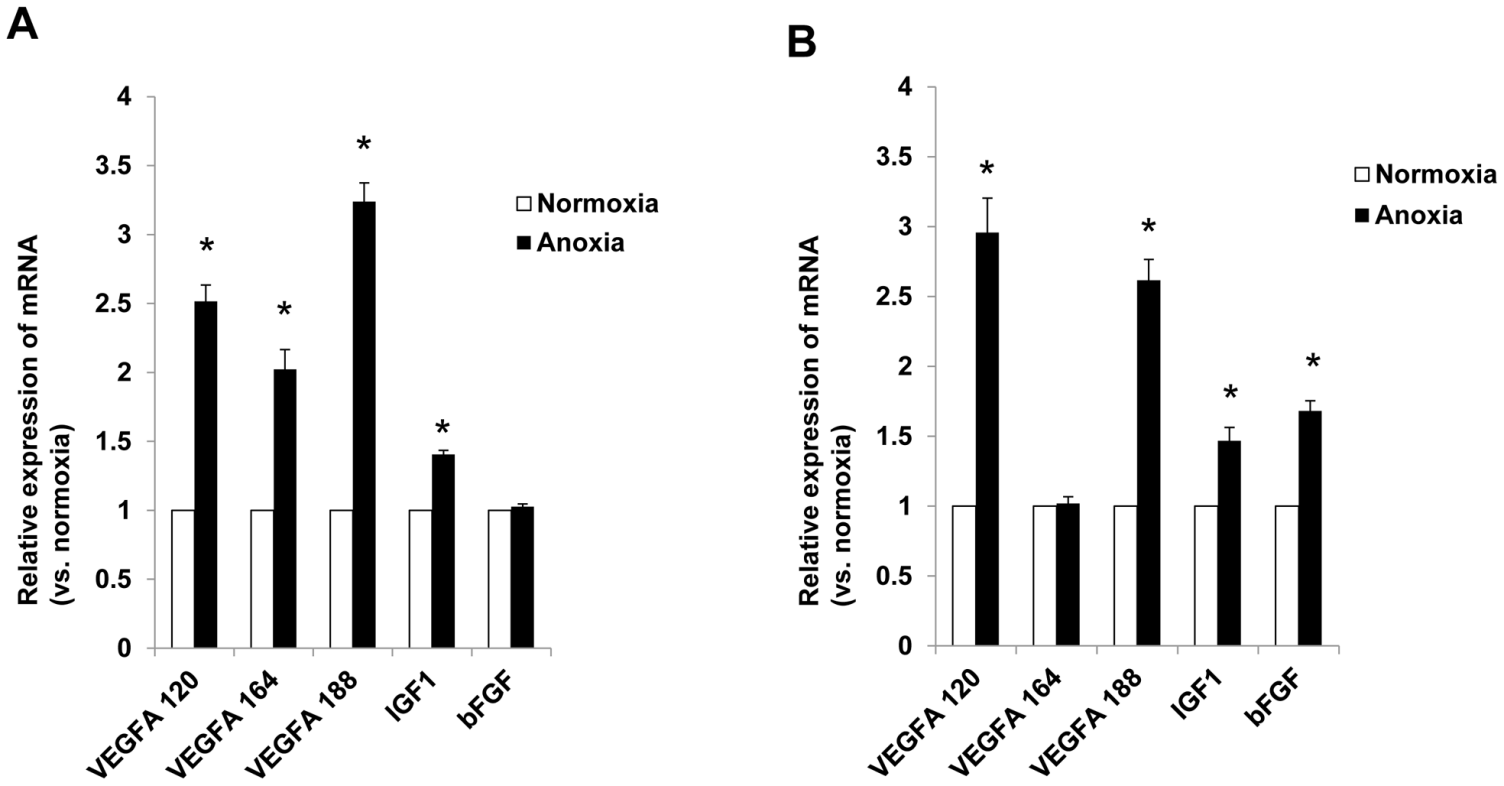
Real-time PCR analysis of mRNA expression of KMSC and KMSC-derived MPs. The relative mRNA expression of vascular endothelial growth factor (VEGF), insulin-like growth factor (IGF), and fibroblast growth factor (FGF) of KMSC (Figure 3A) and their MPs (Figure 3B) in normoxic (white bars) and anoxic (black bars) conditions. Results are expressed as mean ± SE of three different experiments. Kruskal-Wallis test was performed; * P<0.05 versus normoxia control.

Quantitative real time PCR analysis confirmed the relative mRNA expression of proangiogenic vascular endothelial growth factor (VEGF), insulin-like growth factor-1 (IGF-1), and fibroblast growth factor (FGF) in KMSC and KMSC-derived MPs. KMSC-derived MP cDNA was prepared from KMSC cultured in both normoxic and anoxic (<1% O_2_) environments mimicking I/R injury. Compared to normoxic conditions, anoxic conditions induced greater mRNA expression of all VEGF splicing forms (120, 164, 188) and IGF1 in KMSC (Figure 3A). KMSC-derived MPs in anoxic conditions demonstrated significantly higher levels of mRNA expression for VEGF 120, VEGF 188, IGF1, and FGF (Figure 3B).

### Incorporation of KMSC-Derived MPs into HUVEC

**Figure 4:**
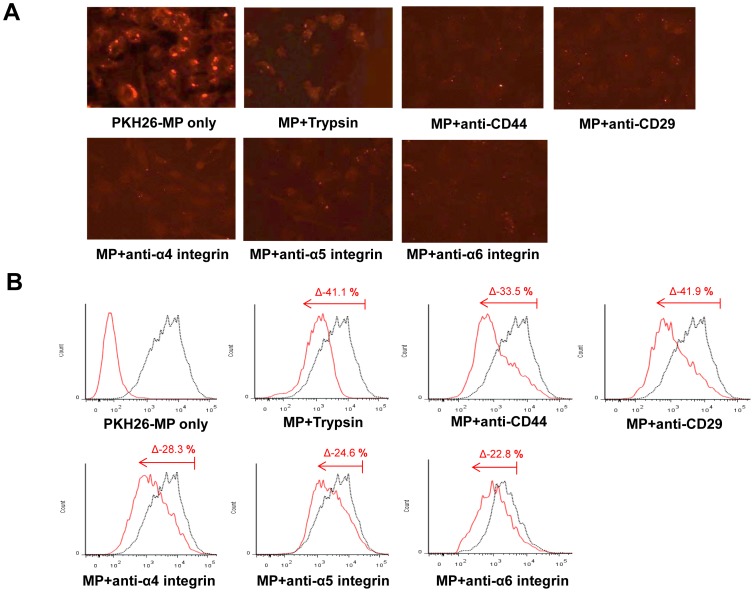
Incorporation of MPs in HUVEC. (A) Immunofluorescence analysis of HUVEC internalization (30 min at 37°C) of MPs labeled with PKH26 preincubated with or without trypsin (0.5 mM), or with 1 µg/ml blocking monoclonal antibody against CD44, CD29, α4 integrin, α5 integrin, and α6 integrin. (B) Representative FACS analyses of HUVEC internalization of MPs labeled with PKH26 (black curves) after 30 min of incubation at 37°C. MPs were preincubated with or without trypsin, or with 1 µg/ml blocking monoclonal antibodies against CD44, CD29, α4 integrin, α5 integrin, and α6 integrin. Black curves indicate internalization of untreated MPs. In the first panel, red curve indicates negative control (cells not incubated with MPs) and black dot curve indicates PKH 26 expression in cultured HUVEC treated with PKH 26 labeled MPs alone. In other panels, red curves indicate internalization of MPs in cultured HUVEC after pretreatment with trypsin or blocking antibodies. Δ denotes the degree of PKH 26 reduction after treatment with trypsin or blocking antibodies compared to PKH 26 labeled MPs alone. Three independent experiments were performed with similar results.

To identify the incorporation of KMSC-derived MPs, endothelial cells (HUVEC) were cultured with MPs labeled with PKH 26 dye. Immunofluorescence microscopy and FACS analysis revealed that PKH 26 labeled MPs were readily incorporated into cultured HUVEC. The incorporation of PKH 26 labeled MPs was significantly inhibited by removing surface molecules with trypsin pretreatment (−41.1±3.7% compared to non-treated control on FACS analysis) (Figure 4). Furthermore, to investigate the role of specific surface molecules, PKH26 labeled MPs were pretreated with 1 µg/ml blocking monoclonal antibodies against CD44, CD29, and α4, 5, and 6 integrins. Pretreatment with anti-CD44, CD29, and α4, α5, and α6 integrin antibodies also significantly inhibited the incorporation of MPs into cultured HUVEC compared to non-treated controls (with anti-CD44: −33.5±4.2%, with anti-CD29: −41.9±0.5%, with anti-α4: −28.3±2.5%, with anti-α5: −24.6±3.3%, with anti-α6: −22.8±5.2%). These results implied that surface molecules such as CD44, CD29, and α4, α5, and α6 integrins all play an important role in the incorporation of KMSC-derived MPs into HUVEC (Figure 4 A, B).

### *In vitro* Cell Proliferation, Anti-apoptotic, and Angiogenic Effects of KMSC-derived MPs

**Figure 5:**
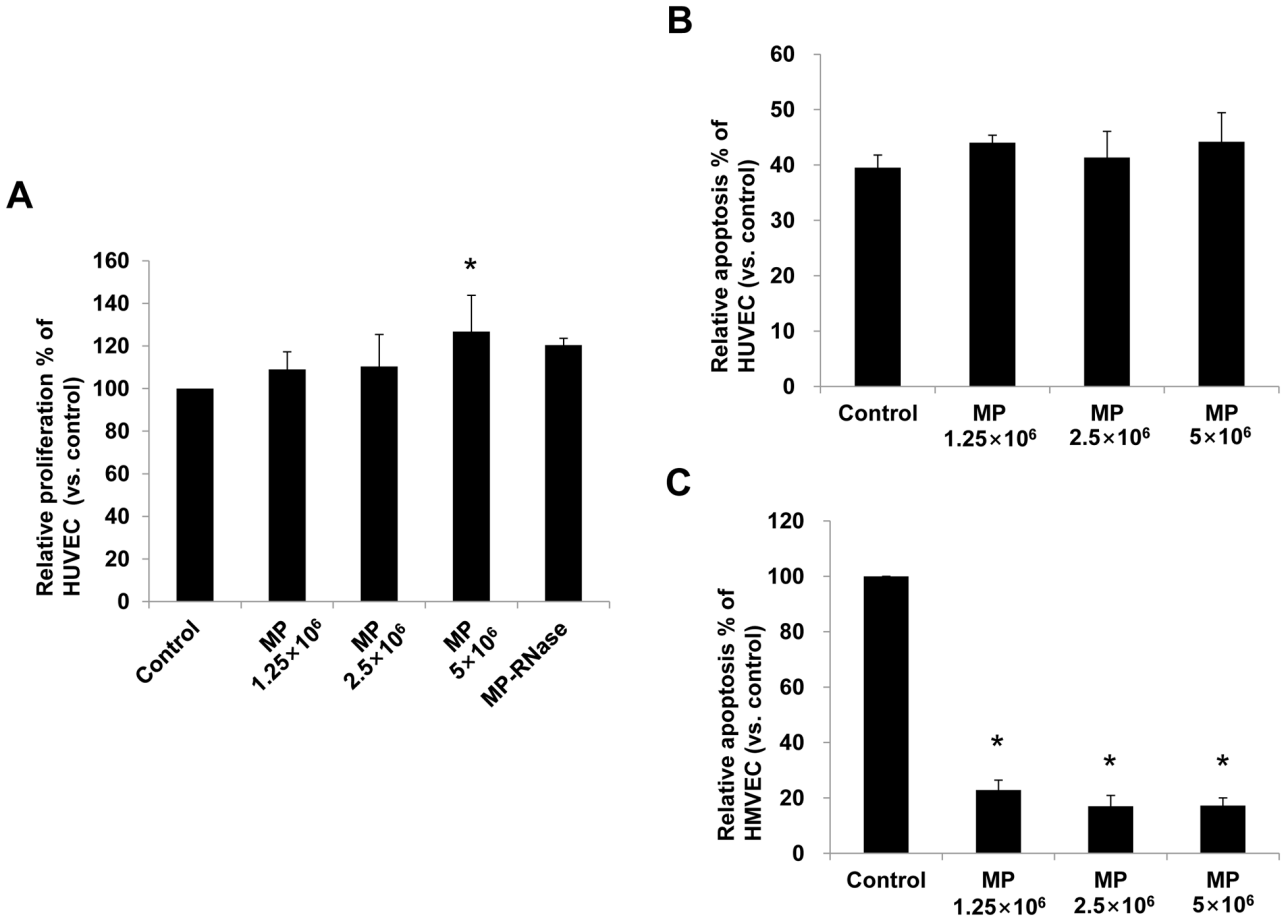
*In vitro* cell proliferation and anti-apoptotic effects of MPs. (A) Proliferation of HUVEC treated with vehicle control, with different doses of MPs or MP-treated with RNase (MP-RNase). (B) Apoptosis assay of HUVEC treated with vehicle control or with different doses of MPs. (C) Apoptosis assay of HMVEC treated with vehicle control or with different doses of MPs. Results are expressed as % compared to vehicle control and mean ± SE of three different experiments. Kruskal-Wallis test was performed; * P<0.05 versus vehicle control.

Incubation of HUVEC with KMSC-derived MPs promoted a significant dose-dependent improvement in HUVEC proliferation in serum deprived culture conditions compared to control cells incubated with vehicle alone (relative degree of proliferation according to MP dose 1.25×10^6^, 2.5×10^6^, 5×10^6^; 109.0±8.3%, 110.3±15.1%, 126.8±17.0% compared to control, Figure 5A). When KMSC-derived MPs were pretreated with RNase that induced complete RNA degradation, the beneficial effect of KMSC-derived MPs on HUVEC proliferation was significantly inhibited (Figure 5A). However, MPs did not inhibit the serum deprivation induced apoptosis of HUVEC *in vitro* (Figure 5B). Heterogeneity and differences between large and small vessel endothelial cells are well known. We therefore, investigated antiapoptotic effect of MPs on HMVEC, which is of microvascular origin. In contrast to HUVEC, apoptosis of HMVEC was significantly inhibited by MPs (relative degree of apoptosis according to MP dose 1.25×10^6^, 2.5×10^6^, 5×10^6^; 22.8±3.6%, 17.0±3.9%, 17.2±2.8% compared to control, Figure 5 C).

**Figure 6:**
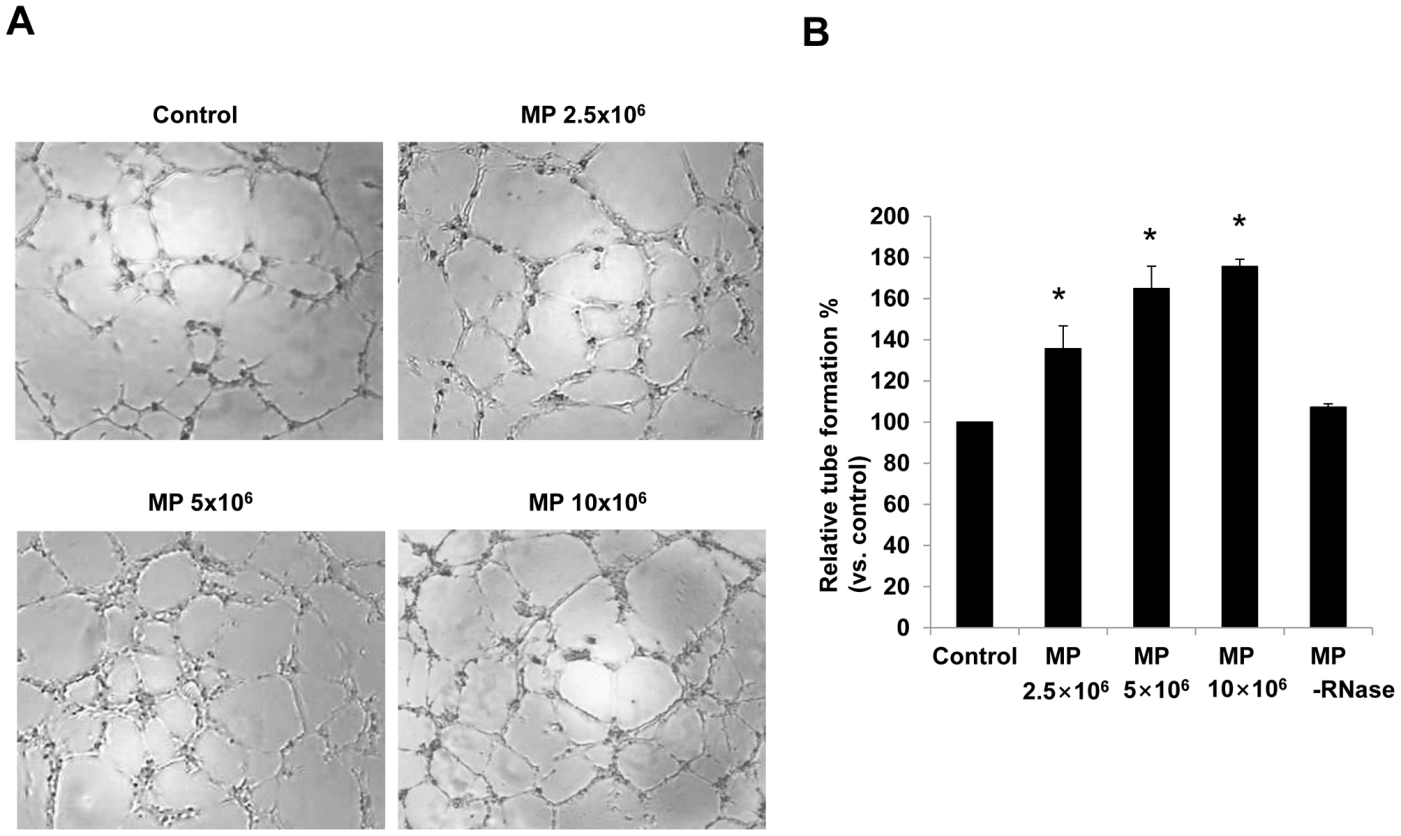
*In vitro* angiogenic effects of MPs. (A) Representative images of angiogenic effects of HUVEC pretreated with vehicle control, with different doses of MPs, or MPs preincubated with RNase (MP-RNase). (B) Quantitative analysis of tube formation of HUVEC was performed. Results are expressed as % versus vehicle alone and mean ± SEM of six different experiments. Results are expressed as mean ± SE of three different experiments. Kruskal-Wallis test was performed; * P<0.05 versus control.

*In vitro* angiogenic effects of KMSC-derived MPs on HUVEC were tested. HUVEC was grown on growth factor reduced Matrigel with or without KMSC-derived MPs. MPs dose-dependently promoted the endothelial capillary tube formation of HUVEC (relative degree of tube formations according to MP dose 2.5×10^6^, 5×10^6^, 10×10^6^; 135.6±11.1, 164.9±10.8, 175.6±3.5% compared to control, Figure 6A and B). This proangiogenic effect of MPs was inhibited by RNase pretreatment (Figure 6B).

### MP-mediated Horizontal mRNA Transfer into HUVEC by *in vitro* and *in vivo* Engraftment of MPs into Peritubular Kidney Capillaries

**Figure 7:**
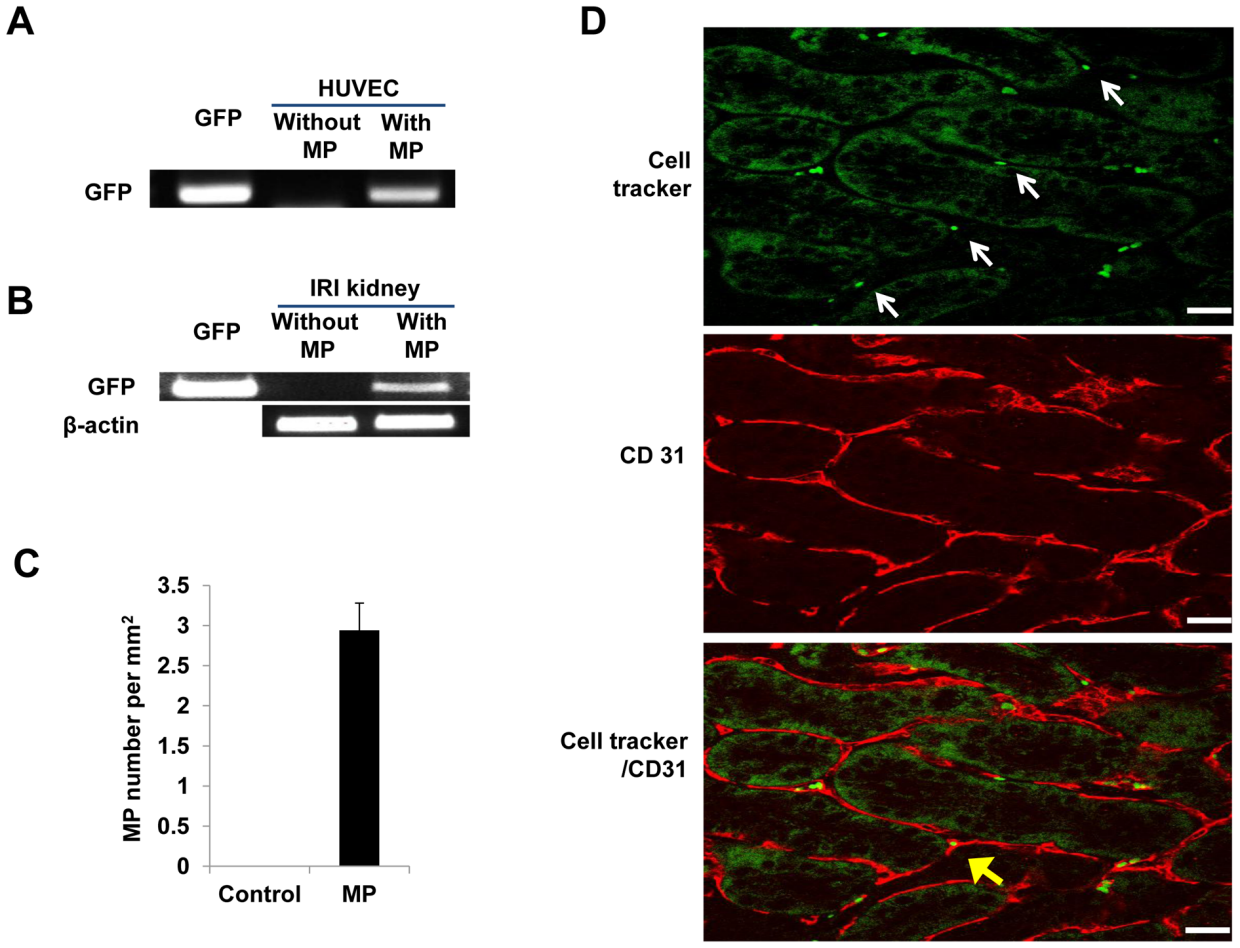
*In vitro* and *in vivo* evidence of MP-mediated horizontal transfer of mRNA into HUVEC and confocal microscopic analysis of engraftment of PKH 26 stained MPs into kidney with I/R injury. (A) RT-PCR analysis for GFP gene in HUVEC. GFP mRNA was detected only from HUVEC incubated with GFP-MPs, and not from untreated HUVEC. (B) Kidney tissue from I/R injured mice that were administered MPs derived from GFP transfected KMSC positively expressed. As a positive control, GFP-DNA was used and β-actin mRNA was used as a housekeeping gene. (C) The average number of engrafted MPs in mice given intravenous injection of MPs from KMSC. MPs were estimated by observing 20 randomly chosen fields from cortex and corticomedullary junction. Results are expressed as mean ± SE. (D) Confocal laser microscopy confirmed green CellTracker™ labeled MPs colocalized within peritubular capillaries stained with red fluorescent anti-CD31 in the kidney. White arrows indicate green CellTracker™ labeled MPs (upper panel). Yellow arrow indicates the engraftment of green CellTracker™ labeled MPs in peritubular capillaries. (lower panel). White bar represents 20 µm.

KMSC was transiently transfected with green fluorescence protein (GFP) DNA to assess horizontal transfer of MP mRNA into target HUVEC. MPs were isolated from KMSC that were transfected with green fluorescence protein DNA (GFP-MP). Untreated control HUVEC do not normally express GFP, as shown by the negative RT-RCR of RNA extracted from control HUVEC. In contrast, GFP mRNA was readily detected in HUVEC incubated with GFP-MPs for 6 hours (Figure 7A). This finding suggests MP-mediated horizontal transfer of GFP mRNA into target HUVEC *in vitro* (Figure 7A). Next, we investigated *in vivo* MP mediated horizontal mRNA transfer in mice kidney tissue. GFP mRNA was expressed only in I/R injury kidney injected with GFP MP via tail vein (Figure 7B).

To examine the engraftment of KMSC-MPs in the kidney, green CellTracker™ labeled MPs were injected into I/R injured FVB/N mice. Mice were sacrificed at day 3 after KMSC-MP injection and the number of average engrafted MPs was 2.9±0.3 MPs/mm^2^ of kidney cortex by semi-quantitative analysis (Figure 7C). Confocal microscope analysis confirmed the engraftment of CellTracker™ labeled MPs in peritubular capillaries expressing CD 31 (Figure 7D).

### KMSC-derived MPs Protect against I/R Injury in Kidneys

**Figure 8:**
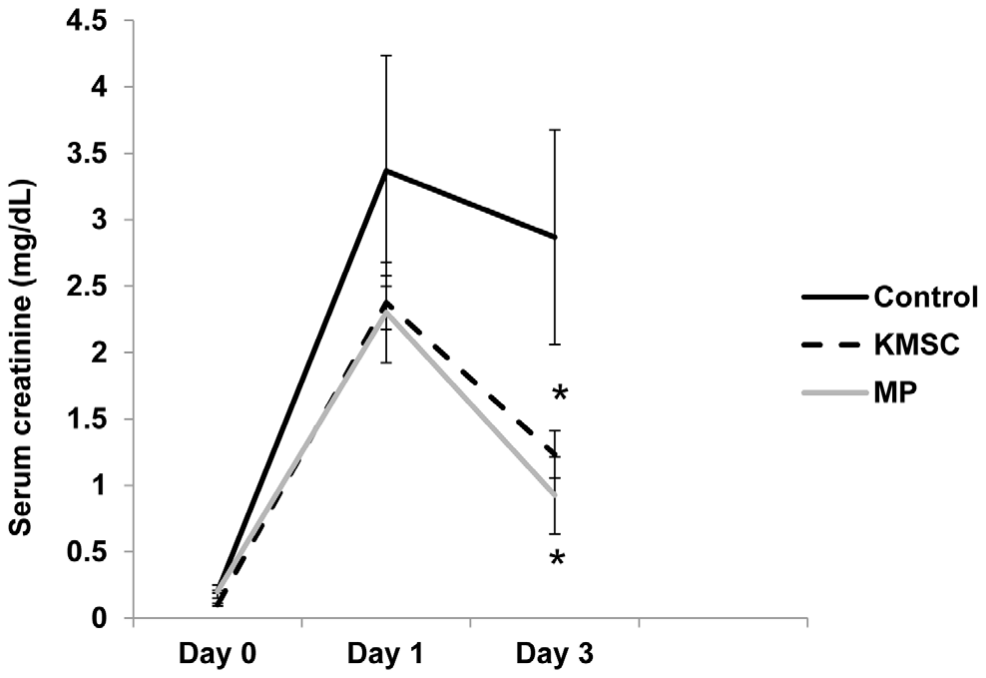
Renoprotective effects of KMSC or KMSC-derived MPs after I/R injury. Mice were given tail vein injections of KMSC, MPs, or vehicle as control on day 3. Plasma creatinine concentration was significantly lower in mice treated with KMSC and KMSC-derived MPs 3 days after I/R renal injury. Results are expressed as mean ± SE of six different experiments. Kruskal-Wallis test was performed; * P<0.05 versus vehicle control.

**Figure 9:**
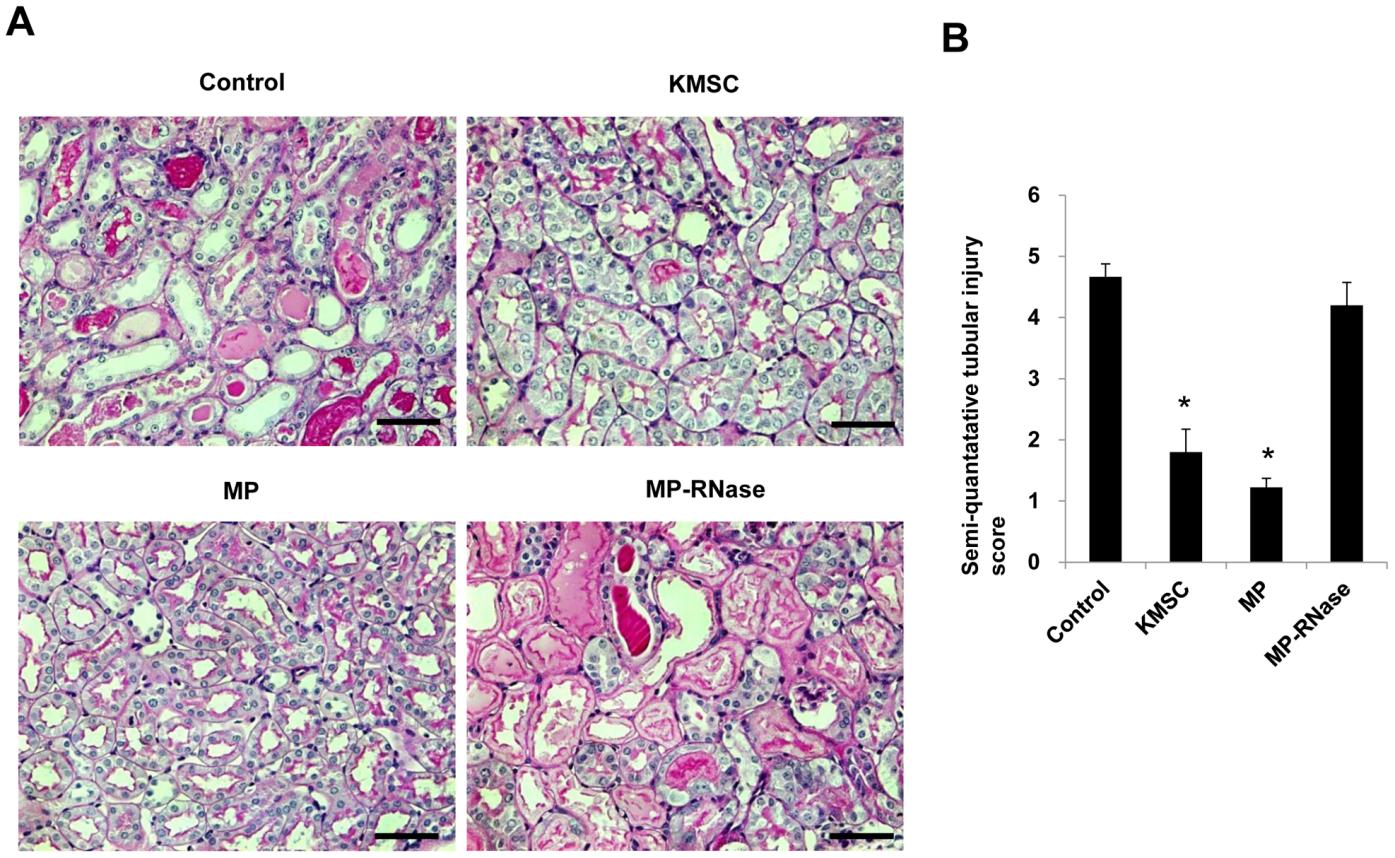
Semi-quantitative injury scoring of I/R injury kidney. (A) Representative images of PAS staining in I/R injury kidney with vehicle control, KMSC, KMSC-derived MPs, and MP-treated with RNase (MP-RNase). (B) Quantitative analysis of I/R injury kidney was determined by semi-quantitative injury scoring (0–5). Injury score was significantly decreased in kidneys injected with KMSC and KMSC-derived MPs compared to those injected with vehicle control or MP-RNase 3 days after IRI. Results are expressed as % versus vehicle alone and mean ± SE of six different experiments. Kruskal-Wallis test was performed; * P<0.05 versus vehicle control. Black bar represents 50 µm.

**Figure 10:**
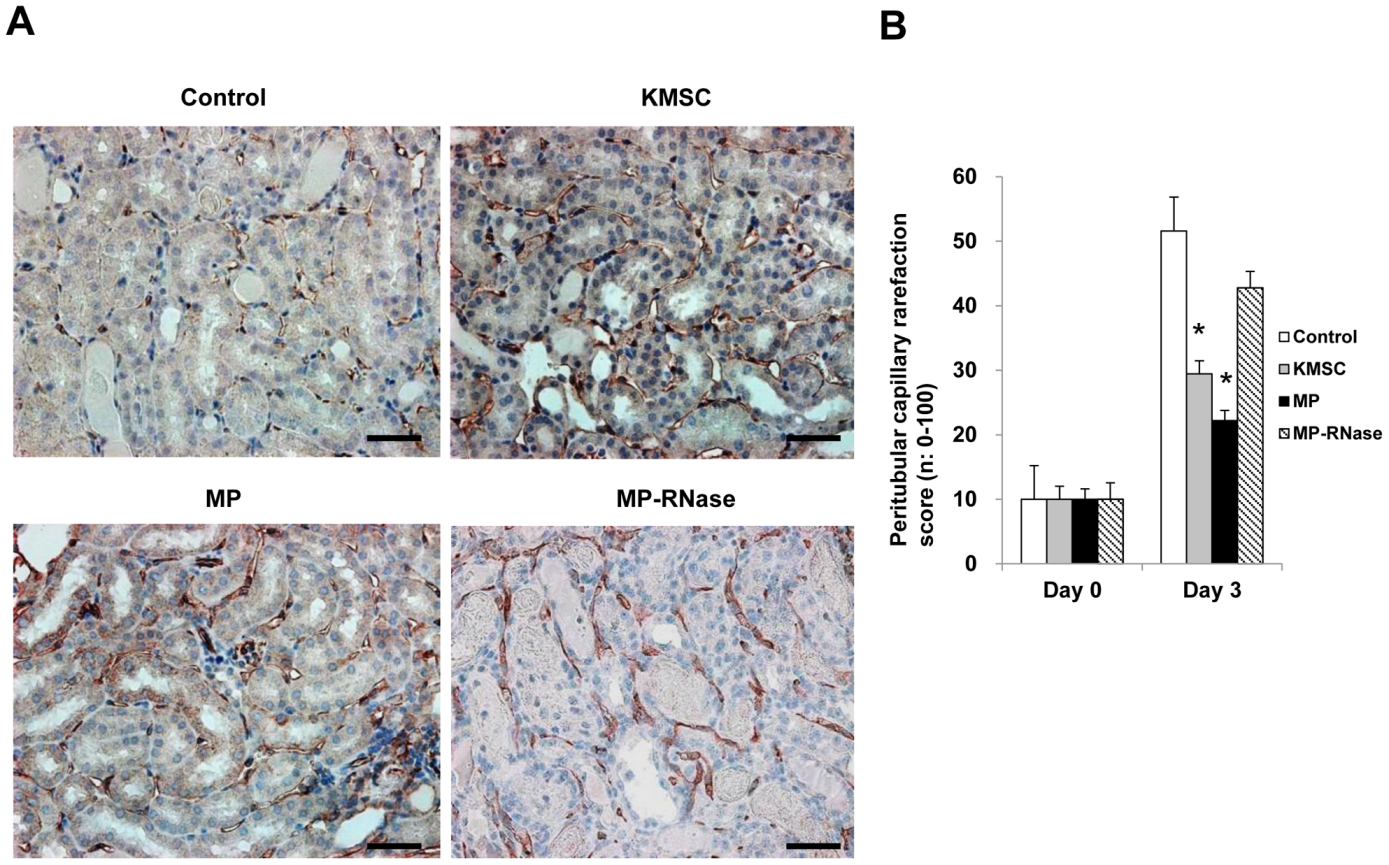
Effects of MPs on microvascular rarefaction of I/R injury kidney. (A) Representative images of CD 31 stainings in I/R injury kidney with vehicle control, KMSC, KMSC-derived MPs, and MP-treated with RNase (MP-RNase). (B) Quantitative analysis of peritubular capillary rarefaction index was determined by CD31 staining. The degree of microvascular rarefaction was significantly decreased in kidneys injected with KMSC and KMSC-derived MPs compared to those injected with vehicle control or MP-RNase 3 days after I/R injury. Results are expressed as % versus vehicle alone and mean ± SE of six different experiments. Kruskal-Wallis test was performed; * P<0.05 versus vehicle control. Black bar represents 50 µm.

Next, the *in vivo* renoprotective effects of KMSC-derived MPs infused in bilateral renal I/R injured mice were investigated. Thirty minutes of bilateral I/R injury induced severe renal dysfunction, as demonstrated by a significant increase in serum creatinine levels. Compared to control ischemic mice, animals that received KMSC-derived MPs as well as KMSC demonstrated significantly lower serum creatinine levels at day 3 (1.2±0.2, 0.9±0.3 vs.2.9±0.8 mg/dL, respectively, Figure 8). This result suggests that KMSC-derived MPs afforded early renoprotection comparable to that of KMSC transplantation, as shown in our previous study. To further explore the mechanism of renoprotection by KMSC-derived MPs, tubular epithelial cell (TEC) morphology and peritubular capillary microvascular density were examined. Kidney sections from ischemic mice treated with KMSC, KMSC-derived MPs, or vehicle and RNase-treated MPs were stained with PAS and processed for CD31 expression, respectively. At day 3, the kidneys of mice injected with vehicle or RNase-treated MPs after I/R injury showed signs of severe tubular lesions by semi-quantitative injury scoring. Mice kidneys with injected MSCs or KMSC-derived MPs showed amelioration of tubular lesions compared to control mice kidneys (4.7±0.2, 4.2±0.8 vs. 1.8±0.4, 1.2±0.1 scores per HPF (high power field) (×400), respectively, P<0.05, Figure 9 A, B). The degree of renal microvascular rarefaction, assessed by CD31 expression, was significantly reduced in mice injected with KMSC-derived MPs at 3 days after I/R injury compared to mice injected with vehicle or RNase-treated MPs (42.8±2.6 vs. 29.5±2.0 or 22.2±1.6 per HPF, respectively, P<0.05) (Figure 10 A, B).

**Figure 11:**
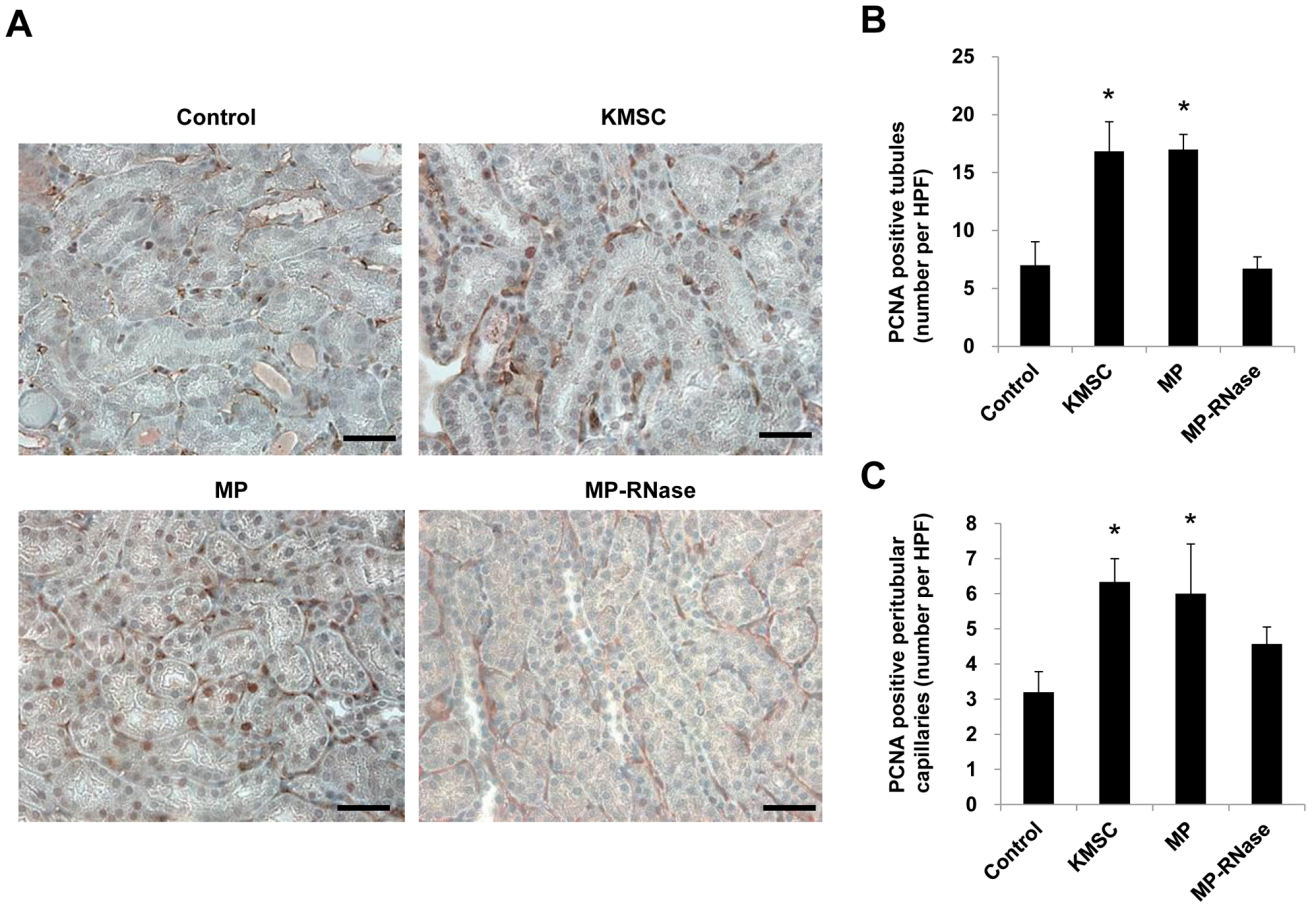
Effects of MPs on tubular epithelial and peritubular capillary endothelial cell proliferation in I/R injury kidney. (A) Representative images of PCNA and CD 31 staining in I/R injury kidney. (B), (C) Quantitative analysis of PCNA-positive cells in kidneys 3 days after I/R injury injury. PCNA-positive counting was used to assess the proliferation of tubular epithelial cells and peritubular capillaries. There were significantly more PCNA-positive tubules (B) and both PCNA and CD 31 positive peritubular capillaries (C) in I/R injury kidneys treated with KMSC and KMSC-derived MPs compared those treated with vehicle control or MP-treated with RNase (MP-RNase) 3 days after I/R injury. Results are expressed as mean ± SE of six different experiments. Kruskal-Wallis test was performed; * P<0.05 versus vehicle control. Black bar represents 50 µm.

To further examine the renoprotective mechanisms of KMSC-derived MPs, the degrees of resident renal cell proliferation and apoptosis were examined in the kidneys of control ischemic and MP-injected mice. PCNA-positive cells were evaluated by counting the number of positive nuclei per field in 10 randomly chosen sections. As shown in Figure 11, the number of PCNA-positive tubular epithelial cells was significantly greater in mice kidneys injected with either KMSC or KMSC-derived MPs compared to mice injected with vehicle or RNase-treated MPs (16.8±2.5, 17.0±1.3 vs. 7.0±2.0, 6.7±1.0 cells per HPF, respectively, P<0.05, Figure 11 A, B). Furthermore, the number of PCNA-positive peritubular capillary endothelial cells was also significantly greater in I/R injured mice kidneys treated with either KMSC and KMSC-derived MPs compared to mice injected with vehicle control or RNase-treated MPs (6.3±0.7, 6.0±1.4 vs. 3.2±0.6, 4.6±0.5 cells per HPF, respectively, P<0.05, Figure 11 A, C).

**Figure 12:**
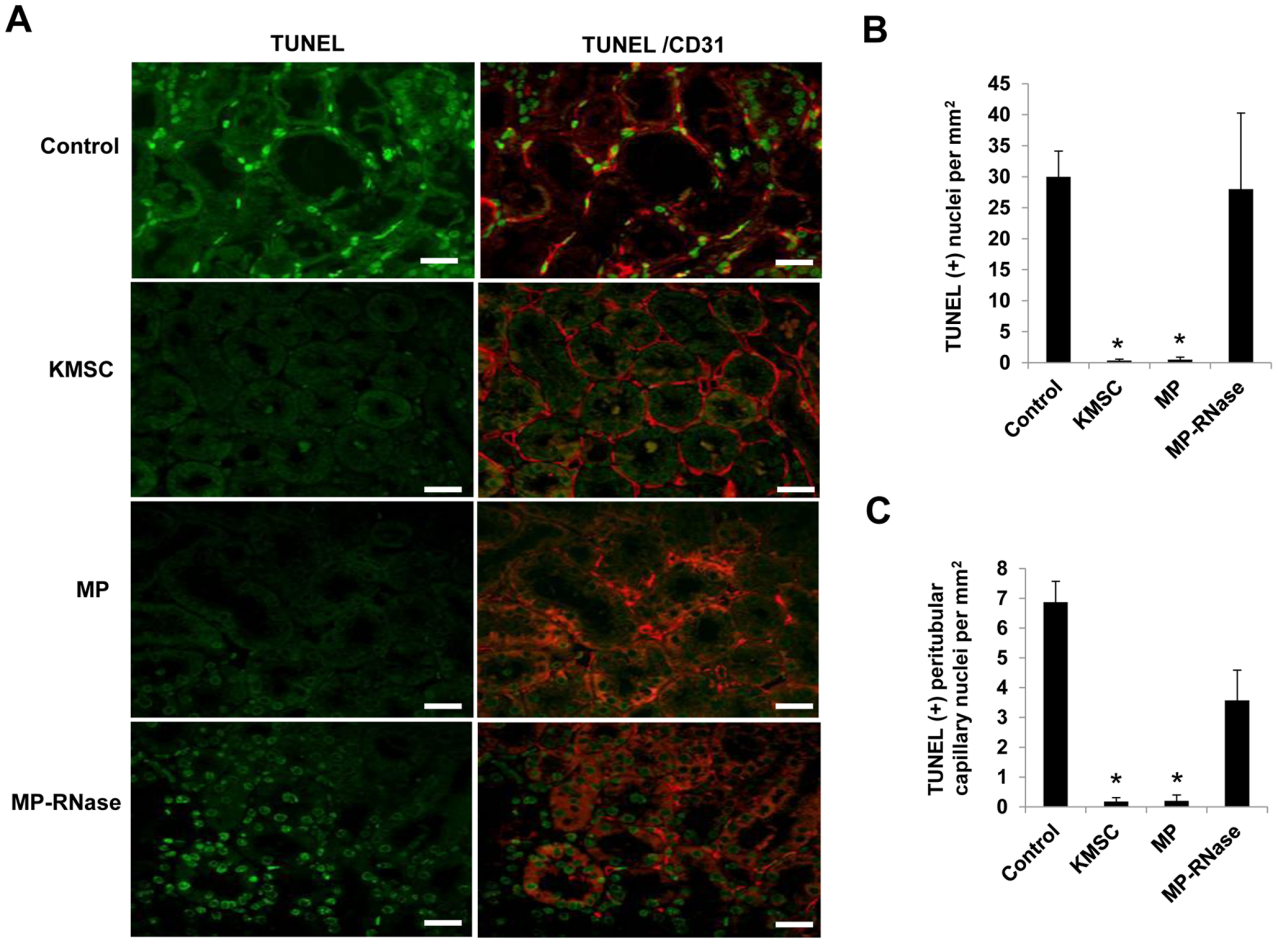
Anti-apoptotic effects of MPs on tubular epithelial and peritubular capillary endothelial cell in I/R injury kidney. (A) Representative confocal microscopic images of TUNEL (green) and TUNEL/CD 31 (red) double staining and (B, C) quantitative analysis of TUNEL-positive cells in the kidney 3 days after I/R injury. The total number of TUNEL-positive nuclei (B) and both TUNEL and CD 31-positive peritubular capillary nuclei (C) significantly decreased in kidneys injected with KMSCs and KMSC-derived MPs compared to those injected with vehicle control or MP-treated with RNase (MP-RNase) 3 days after IRI. Results are expressed as mean ± SE of six different experiments. Kruskal-Wallis test was performed; * P<0.05 versus vehicle control. White bar represents 20 µm.

The anti-apoptotic effects of MPs on I/R injured kidneys were evaluated by TUNEL staining. At day 3 after I/R injury, there were significantly fewer TUNEL positive apoptotic TECs in kidneys of ischemic mice injected with either KMSC or KMSC-derived MPs compared to those injected with vehicle or RNase-treated MPs in confocal microscopic analysis (0.4±0.2, 0.5±0.4 vs. 30.0±4.1, 28.0±12.3 cells per HPF, respectively, P<0.05) (Figure 12 A, B). The apoptotic peritubular capillary endothelial cells that expressed CD 31 were simultaneously measured with TUNEL. The total number of TUNEL positive peritubular capillary endothelial nuclei was also significantly reduced in kidneys of ischemic mice treated with KMSC or KMSC-derived MPs compared to mice injected with vehicle control or RNase-treated MPs (0.2±0.1, 0.2±0.2 vs.6.9±0.7, 3.6±1.0 cells per HPF, respectively, P<0.05) (Figure 12 A, C).

These data indicate that in I/R injured mice, injection of KMSC-derived MPs afforded comparable renoprotective effect to injecting KMSC alone. This was achieved not only by an effect on TECs but also by stimulating the proliferation and inhibiting the apoptosis of peritubular capillary endothelial cells.

## Discussion

The present study focused on characterizing the proangiogenic potential of KMSC-derived MPs and their contribution to peritubular capillary repair after ischemic injury via horizontal transfer of genetic materials. The biological characteristics of MPs are believed to be dependent on activation stimuli or the microenvironment in which MPs are produced. [2] KMSC in present study were cultured in serum-free MEM for 24 hours in a hypoxic chamber to mimic the microenvironment of acute I/R injury. KMSC-derived MPs generated in this anoxic culture condition demonstrated significantly higher levels of VEGF-A, IGF-1 and FGF mRNA compared to those released in normoxic culture condition. These results implied that there were possible different properties of MP produced in the ischemic environment compared to normal, non-stressed conditions. Such increased expression of proangiogenic MP transcripts in anoxic conditions may contribute to more efficient regeneration of injured organs and is clinically relevant as more MPs with proangiogenic characteristics are needed in time of tissue repair.

VEGF-A is a major player in angiogenesis and is involved in matrix remodeling, adhesion molecule expression and stimulates the proliferation of peritubular capillaries that are pivotal for tubular epithelial cell regeneration. It is alternatively processed in at least three isoforms in the mouse, which differ in receptor susceptibility, mitogenic activity, and tissue specific expression. [23] KMSC-derived MPs generated in anoxic condition showed all three isoforms with significant increases in the cell-associated isoform of VEGF-A 188 and its cleaved NH2-terminal fragment (VEGF-A 120) by real time PCR analysis. [24] An overall inhibition of the renal VEGF pathway has been observed during the early time points following I/R injury. The expression of renal VEGF mRNA is significantly repressed possibly impairing initial renal vasculature repair and thus contributing to persistent rarefaction of renal blood vessels. In a rat model of renal ischemia/reperfusion injury, VEGF mRNA and protein were not increased. Instead, preexisting VEGF in the tubular cell cytoplasm redistributed to the basolateral surface thus allowing closer access to injured endothelial cells. Therefore, delivery of VEGF-A mRNA via exogenous MPs administration during early post-ischemic period may contribute to observed *in vivo* improvements in renal function and histology, as evidenced by lower serum creatinine and peritubular capillary rarefaction index. Furthermore, it has been postulated that the differential localization and balance of proangiogenic growth factors influence the behavior of the responding endothelium. KMSC-derived MPs also showed the increased IGF1 and bFGF mRNA, both of which are important in sustaining stem cell-mediated renal repair and angiogenesis, suggesting that these MPs are suitable agents of renoprotection in I/R injury.

Several studies have highlighted renoprotective effects of administration of MSC as well as MPs derived from bone marrow MSC in renal injury models. [13]–[15] The results mainly showed increase in proliferation or attenuation of tubular epithelial cells apoptosis. However, little attention was given to rarefaction of renal vasculature endothelial cells following the I/R injury. Net shift of growth factors in favor of angiogenesis early post-ischemic I/R setting may result in vasodilatation and reduction of vasoconstriction that is important for oxygen delivery to viable cells and waste product removal from injured tissues. KMSC-derived MPs stained with PKH26 were found engrafted near the peritubular capillary area, which in some sections coincided with CD31 positive cells. KMSC-MP infusion immediately after I/R injury induced proliferation and inhibited apoptosis of not only tubular epithelial cells but also peritubular capillary endothelial cells, resulting in better preservation of the peritubular microvasculature. Our data are in accordance with a recent study in ischemic rats that reported improved renal microvascular rarefaction and preservation of renal function after administration of EPC-derived MPs. These EPC-derived MPs are well known to express mRNA and microRNA rich in proangiogenic factors. [16] Several studies have demonstrated that MPs of stem cell origin confer renoprotective effects in rodent models of AKI via transfer of messenger RNA and microRNA to target cells. Pretreatment of MPs with high concentrations of RNase inactivates mRNAs or RNAs carried by MPs. We also observed that pretreatment with RNase significantly reduced the *in vitro* as well as *in vivo* effects of MPs on functional as well as histological alterations of peritubular capillary endothelial cells induced by I/R injury. These findings suggest that the horizontal transfer of proangiogenic transcripts from KMSC-derived MPs to resident renal cells is instrumental in the renoprotective actions of MPs. Effective mRNA transfer from KMSC-derived MPs to target endothelial cells was evidenced by GFP mRNA expression in GFP-MP treated HUVEC and GFP-MP injected mice kidney with I/R injury.

Another interesting finding of this study is that the *in vitro* anti-apoptotic effects of KMSC-derived MPs were different between the two endothelial cell types, HUVEC and HMVEC. Endothelial cells from small capillaries differ from those of large veins in their nutritional requirements and responses to growth stimuli as well as their angiogenic potential. KMSC-derived MPs significantly reduced apoptosis of HMVEC, which originates from human microvasculature, in accord with in vivo findings of decreased peritubular capillary endothelial cell apoptosis in IRI mice treated with MPs. HUVEC show different characteristics in the production of angiogenic growth factors, extracellular matrix composition, and proliferation compared to human microvascular endothelial cells. [25], [26] HMVEC have been reported to differ from HUVEC in the expression of chemokine receptors and other surface markers. [27], [28] Recent study showed different proteome profiles between HUVEC and HMVEC, which could result in distinct biological properties and explain their different responses to KMSC-MPs [29].

In conclusion, the *in vivo* and *in vitro* findings of this study suggest a possible role of KMSC-derived MPs as mediators of renoprotection that is similar to the paracrine mechanism of renoprotection observed with KMSC administration in our previous study.

Further studies on the mechanism of proangiogenic MPs formation and factors determining their biologic characteristics are needed to refine the future therapeutic potential of MPs in acute renal ischemia.
